# Hyperglycosylated-hCG: Its Role in Trophoblast Invasion and Intrauterine Growth Restriction

**DOI:** 10.3390/cells12121647

**Published:** 2023-06-16

**Authors:** Catalin Gabriel Herghelegiu, Alina Veduta, Miruna Florina Stefan, Stefania Lucia Magda, Iuliana Ionascu, Viorica Elena Radoi, Daniela Nuti Oprescu, Alina Mihaela Calin

**Affiliations:** 1 Institutul National pentru Sanatatea Mamei si a Copilului “Alessandrescu Rusescu”, 020395 Bucharest, Romania; 2 Filantropia Hospital, 011171 Bucharest, Romania; alina.veduta@gmail.com; 3 Department of Cardiology, University and Emergency Hospital, 050098 Bucharest, Romania; stefan_miruna@yahoo.com (M.F.S.); stefaniamagda@yahoo.com (S.L.M.); 4 Department of Cardiology and Cardiovascular Surgery, University of Medicine and Pharmacy Carol Davila, 020021 Bucharest, Romania; 5 Faculty of Veterinary Medicine, University of Agronomical Sciences and Veterinary Medicine, 011464 Bucharest, Romania; iuliana.ionascu@usamv.ro; 6 Department of Genetics, University of Medicine and Pharmacy Carol Davila, 020021 Bucharest, Romania; viorica.radoi@yahoo.com; 7 Department of Obstetrics and Gynecology, University of Medicine and Pharmacy Carol Davila, 020021 Bucharest, Romania; 8 Medicine and Pharmacy Faculty, Dunarea de Jos University, 800008 Galati, Romania; alina.calin@ugal.ro

**Keywords:** hyperglycosylated hCG, fetal growth restriction, trophoblast invasion, uterine arteries

## Abstract

Human chorionic gonadotropin (hCG) is produced by the placenta and its roles have been studied for over a century, being the first known pregnancy-related protein. Although its main role is to stimulate the production of progesterone by corpus luteal cells, hCG does not represent just one biologically active molecule, but a group of at least five variants, produced by different cells and each with different functions. The hyperglycosylated variant of hCG (H-hCG) plays a key role in trophoblast invasion, placental development and fetal growth. During trophoblast invasion, H-hCG promotes extravillous cytotrophoblast cells to infiltrate the decidua, and also to colonize and remodel the spiral arteries in to low resistance, larger-diameter vessels. As fetal growth is heavily reliant on nutrient availability, impaired trophoblast invasion and remodeling of the uterine arteries, leads to a defective perfusion of the placenta and fetal growth restriction. Understanding the function of H-hCG in the evolution of the placenta might unveil new ways to manage and treat fetal growth restriction.

## 1. Introduction

Human chorionic gonadotropin (hCG) is produced by the placenta and its roles have been studied for over a century. It is the first known pregnancy-related protein. In 1920, Hiroese first discovered a link between hCG and progesterone production by corpus luteal cells [1]. Aschheim and Zondek identified hCG in the urine of pregnant women and developed the first pregnancy test in 1927 [2]. For many years, hCG was thought of as one molecule: a hormone with the sole function to stimulate the production of progesterone by corpus luteal cells, and by doing so, helps to maintain the pregnancy. In recent decades, there been a change of view with the discovery that hCG does not represent just one biologically active molecule, but a group of at least five variants, produced by different cells and each with different functions [3]. This paper focuses on the hyperglycosylated variant of hCG and its roles in trophoblast invasion, placental development and fetal growth.

## 2. Structure and Variants of hCG

As we know today, hCG is a 237 amino acid heterodimer, composed of an α-subunit, which is common to hCG, TSH, LH, and FSH and a β-subunit, which gives the biological specificity of the hormone. The two subunits are held together by non-covalent hydrophobic and ionic interactions [4,5]. The β-subunit of LH consists of 121 amino acids and the hCG β-subunit consists of 145 amino acids, which makes them quite similar. Hence, it is noteworthy that the hCG β-subunit and LH β-subunit exhibit a remarkable degree of protein sequence similarity (>85%) and possess close immunological resemblance. As a result, hCG binds to the LH receptor, stimulating corpus luteal cells to produce progesterone [5]. The primary structural distinction between the hCG β-subunit and LH β-subunit lies in the presence of a carboxyl-terminal peptide extension in the hCG β-subunit, known as hCGβCTP, which spans amino acids 113 to 145. Antibodies that specifically recognize epitopes within hCGβCTP are extensively employed in highly specific hCG assays [6].

Research has shown that hCG is the most heavily glycosylated glycoprotein of the group. It is estimated that between 28% to 39% of the molecule’s weight is attributed to its sugar side-chains [3]. The intricate structure of hCG allows for molecular heterogeneity in both its protein and carbohydrate components, resulting in five distinct molecules that have different functions. The first three, “regular” hCG, hyperglycosylated hCG (H-hCG), and sulfated hCG, are dimer variants and have the same amino acid structure but differ in carbohydrate structure. The last two, β-hCG and hyperglycosylated β-hCG, are β-subunit monomer variants with different carbohydrate structures, and have been detected in non-trophoblastic advanced malignancies [3,7,8,9,10].

H-hCG is a super-glycosylated variant of hCG with four double-molecular-sized hexasaccharide O-linked sugars and four triantennary N-linked sugars [9,11]. The molecular weight is increased to 42,800 due to the presence of added sugars, which account for 39% of its total weight [12]. The structural similarities between H-hCG and transforming growth factor-β (TGF-β) suggest that H-hCG may be folded differently compared to “regular” hCG due to the added sugars. These structural changes expose a central cysteine knot structure that allows it to bind to the TGF-β receptor [13]. By doing so, H-hCG acts as an antagonist to the TGF-β receptor, promoting growth and invasion [3,5,14]. Additionally, although still debated, some studies suggest that H-hCG also activates the classic LH receptor, promoting progesterone production, albeit to a lesser extent than hCG [14].

## 3. hCG and H-hCG Production

After fertilization, hCG is one of the first molecules secreted by the primitive trophoblast as implantation occurs. Following implantation, the first trophoblast signal detected in maternal blood is hCG, explaining why it is used to diagnose early pregnancy [15].

Transcription and secretion of hCG begins at the early stages of blastocyst development. By using RT-PCR, it was detected that hCG mRNA expression begins as early as the two-cell stage of embryo development and it increases steadily towards the blastocyst stage [16]. The blastocyst starts producing the protein before implantation, around day 7, and peak concentrations are observed towards day 10 [16,17]. After, implantation production of hCG is mainly attributed to the syncytiotrophoblast and it reaches its peak during the first trimester of the pregnancy around the 10 weeks of gestation [18]. Although it has been well established that the site of hCG production is the villous syncytiotrophoblast, the discovery of the site of H-hCG production is a relatively recent development [19,20,21]. G Kovalevskaya et al. were the first to discover that the H-hCG variant is produced by cytotrophoblast cells, and only recently have researchers identified that H-hCG is specifically produced by extravillous cytotrophoblast cells [15,22]. During trophoblast invasion, extravillous cytotrophoblast cells infiltrate the decidua, anchoring the placenta to the uterus. Extravillous cytotrophoblast cells also participate in the endovascular invasion and infiltrate even deeper, reaching the inner third of the myometrium and colonize the spiral arteries [23,24,25]. Thus, the site of H-hCG production depends on where the extravillous cytotrophoblast cells are located.

The synthesis and secretion of H-hCG involves various enzymes, including glycosyltransferases, proteases, sulfotransferases, and glycosidases. These enzymes add sugar moieties, process the hCG β-subunit, and modify sugar structures attached to hCG [26]. The exact synthesis process is not fully described in the literature. Still, one key enzyme, Core2 β1,6-N-acetylglucosaminyl transferase, is thought to be responsible for hyperglycosylating hCG, leading to the formation of H-hCG variants with altered properties [26,27]. Moreover, studies showed that N-Acetylglucosaminyltransferase (GnT) has been linked to cellular transformation and metastatic potential in cancer cells [26,28]. Immunohistochemistry studies revealed a strong GnT expression in the cytoplasm of extravillous trophoblasts in the anchoring villi. Reducing the GnT expression in choriocarcinoma cells enhanced their migration, invasive capacity, and adhesion to extracellular matrix proteins [29,30].

The production of H-hCG differs greatly depending on the stage of pregnancy. During the initial 3–4 weeks of gestation, H-hCG is the primary form of hCG and accounts for 90% of total hCG [11,21]. In the first trimester, production of H-hCG steadily rises and peaks at around 10 weeks of gestation. However, as pregnancy progresses, the concentration of H-hCG rapidly declines and by the end of the second and, during the third trimester, it represents only 1% of the total hCG [21]. To measure H-hCG levels in the blood of pregnant women, Birken et al. have developed a monoclonal antibody called B152, initially identified in a choriocarcinoma patient [31]. This antibody specifically targets hCG-H and does not cross-react with hCG or its subunits making it ideal for determining H-hCG blood values [11,31]. By utilizing the monoclonal B152 antibody, an assay for hyperglycosylated hCG has been developed and successfully employed as a screening test for Down’s syndrome during the second trimester [32].

## 4. Role of H-hCG in Trophoblast Invasion

During the first trimester, the invasive extravillous cytotrophoblast cells are responsible for the production of high levels of H-hCG, representing the dominant variant of hCG at this time [11,21]. It is believed that the H-hCG is an autocrine factor, as it binds and antagonizes the TGF-β receptors found on extravillous cytotrophoblast cells, enhancing their invasive capabilities [3,5,14]. TGF-β, a powerful regulator of cell proliferation, acts as a tumor suppressor in normal circumstances. However, in tumor cells, TGF-β loses its anti-proliferative effect and instead becomes an oncogenic factor. The TGF-β type 3 co-receptor (betaglycan) probably plays a central role, as it has been demonstrated to be a regulator of human trophoblast differentiation toward an invasive phenotype [33]. The expression of TGF-β3 falls significantly around 9 weeks, precisely at the time of maximal trophoblast invasion, confirming its role as an antagonist of cell migration and invasion [34]. The presence of H-hCG in early pregnancy serum and urine, as well as in choriocarcinoma serum and urine, provides further evidence that it is involved in pregnancy implantation and invasive conditions, such as choriocarcinoma [11]. These observations support the idea that H-hCG plays a major in role in promoting trophoblast invasion. Some studies go even further, suggesting that H-hCG promotes cell growth in choriocarcinoma, as well as other advanced cancers, by antagonizing the TGF-β3 receptor [10,35,36]. Further studies have shown that only the H-hCG variant secreted by extravillous cytotrophoblast cells and not “regular” hCG secreted by syncytiotrophoblast cells, promotes trophoblast invasion [11,15,37].

**Figure 1:**
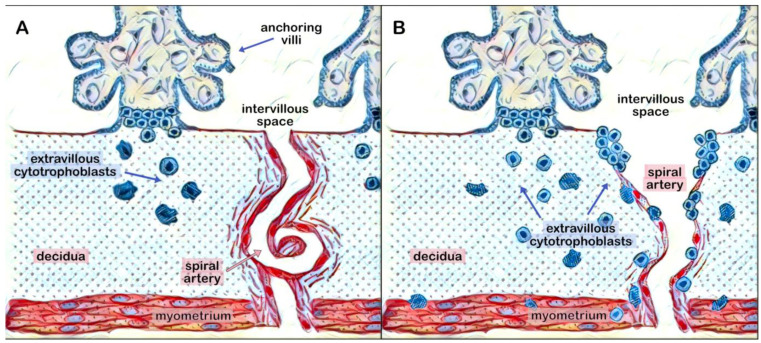
Schematic representation of extravillous cytotrophoblasts cells penetrating the decidua up to the myometrium and infiltrating the uterine spiral arteries, beginning from the first weeks of pregnancy (**A**) until normal remodeling of the arteries is completed (**B**).

**Figure 2:**
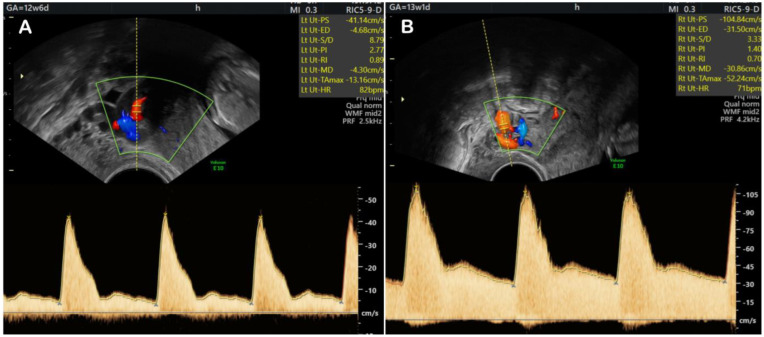
Doppler evaluation of uterine arteries at the end of the first trimester. (**A**) The Doppler profile of the uterine artery in the case of impaired invasion and remodeling of the spiral arteries (or before pregnancy), with increased RI and PI characteristic for high-resistance, small-diameter vessels. (**B**) The Doppler profile of the uterine artery during pregnancy, after normal invasion and remodeling of the spiral arteries, with decreased RI and PI characteristic for low-resistance, large-diameter vessels.

After implantation, extravillous cytotrophoblasts cells are situated at the end of the anchoring villi, in contact with the uterine wall. Under the apocrine influence of H-hCG, the extravillous cytotrophoblasts cells proliferate and form columns of cells, which then penetrate the decidua and rise to the upper third of the myometrium (Figure 1) [15]. In addition, some of the extravillous cytotrophoblasts cells infiltrate the uterine spiral arteries through endovascular or perivascular routes, forming an endovascular trophoblast that replaces the endothelial lining and much of the musculoelastic tissue. This results in widening and the loss of vasomotor contractility of the vessels (Figure 1). This process of invasion and remodeling transforms the uterine spiral arteries from high-resistance, small-diameter vessels to low-resistance, larger-diameter vessels (Figure 2); a process crucial for providing a greater supply of blood to the intervillous spaces, required for fetal growth [23].

## 5. Fetal Growth Restriction

Fetal growth restriction (FGR) is a condition where the fetus fails to reach its genetically predetermined growth potential [38]. A common method of defining fetal growth restriction is when the estimated fetal weight (EFW) falls below the 10th percentile of the given reference ranges that are specific to gestational age [39,40]. Although, based on this definition, the expected prevalence of fetal growth restriction should be approximately 10%; the actual incidence varies based on the population and country, with developing and developed countries having different rates [41,42]. Additionally, some cases of fetal growth restriction may not be detected or reported, and some cases considered to be fetal growth restriction may just be constitutionally small fetuses. It is important to differentiate between fetuses with growth restriction and constitutionally small fetuses, as the first have a high risk for adverse perinatal outcome [43].

Diagnosis of fetal growth restriction is often not straightforward, because fetal growth and weight cannot be evaluated directly by ultrasound measurement of fetal size, and the growth potential is hypothetical. The most common way of calculating the EFW is by using various formulae that consider biometric measurements of the head circumference, biparietal diameter, abdominal circumference and femur length, but numerous alternatives have been proposed [44,45,46]. Despite its widespread use, using these formulae to calculate the EFW also has some drawbacks. The biggest disadvantage is that errors in single-parameter ultrasound measurements are amplified, leading to potentially significant discrepancies in estimated fetal weight. Furthermore, most fetal weight prediction models take into account the abdominal circumference, an ultrasound parameter that can be difficult to measure due to technical factors. Thus, the accuracy of estimated fetal weight can be affected by substantial intra- and inter-observer variability, with errors of approximately 10–15% being commonly reported [47]. Prior to the Delphi consensus criteria published in 2016, the most commonly used definition of FGR was based on assessing the size of the fetus, using EFW or abdominal circumference, and comparing it to specific reference ranges or standards. However, the current definition of FGR now includes additional diagnostic criteria, such as functional parameters, which can provide information about placental dysfunction. Specifically, uterine artery increased Resistive Index (RI) and Pulsatility Index (PI) (Figure 2), above the 95th percentile, can indicate impaired trophoblast invasion and placental dysfunction, and is now included in the definition of FGR [43].

Fetal growth is heavily reliant on nutrient availability, which in turn is influenced by maternal diet, uteroplacental blood supply, placental villous development and, ultimately, the capacity of the villous trophoblast and fetoplacental circulation to absorb and transport these nutrients. The spiral arterioles that perfuse the intervillous space, undergo significant morphological changes during pregnancy and transform into low-resistance, dilated vessels that can sustain an increased blood flow and meet the needs of the fetoplacental unit. Failure to transform these vessels is associated with increased uterine RI and PI, leading to a defective perfusion of the placenta [48,49]. When faced with hypoxia and a reduced supply of nutrients, the fetus makes adjustments to match its growth with the available energy. Thus, a reduced supply of nutrients and oxygen for the fetus is the most common cause of fetal growth restriction [40,50,51,52]. The maternal cardiovascular system also undergoes compensatory changes in an attempt to increase the blood flow to the placenta, which in turn commonly determines maternal gestational hypertension and preeclampsia [53]. In summary fetal growth restriction, gestational hypertension and preeclampsia are all closely related complications that occur in the latter stages of pregnancy and are caused by inadequate cytotrophoblast invasion in the early stages of the pregnancy.

FGR has been linked to an increased risk of perinatal mortality and morbidity, as well as long-term adverse outcomes in infants. Neonates with growth restriction have higher rates of cerebral palsy, (four to six times greater than normally grown fetuses) and have worse neurodevelopmental outcomes [54]. Other neonatal morbidities associated with growth-restricted fetuses include polycythemia, hyperbilirubinemia, and hypoglycemia [55]. Studies on long-term outcomes have demonstrated associations between low birth weight and hypertension, metabolic syndrome, insulin resistance, type-2 diabetes mellitus, coronary heart disease, and stroke [55,56]. In addition, FGR is the second leading cause of perinatal mortality, after prematurity and up to 30–40% of stillbirths are linked to FGR [41,52,56,57].

## 6. Future Directions

FGR remains a challenging issue in modern obstetrics, resulting in elevated fetal and neonatal morbidity and mortality. Although various risk factors have been identified, the cellular and molecular mechanisms underlying its development are still under discussion. Studies using experimental animal models that simulate FGR could offer further insight into the role of H-hCG in trophoblast invasion. In recent years, due to its similarities in fetal development and placental physiology to humans, the rabbit has gained recognition as a valuable experimental animal. Much like in humans, the rabbit placenta is hemochorial, meaning that maternal blood comes into direct contact with trophoblast cells. Studies on rabbits have shown that trophoblast cells participate in the remodeling of the maternal spiral arteries, but to a lesser extent than that in humans [58]. The guinea pig is considered the most suitable animal model for investigating the causes of insufficient spiral artery remodeling and mechanisms of FGR. This is because guinea pigs exhibit extensive trophoblast invasion, which extends deep into the spiral artery walls [57].

Research indicates that H-hCG has a significant role in trophoblast invasion and can serve as a potential predictive marker for FGR onset. Additionally, the abnormal transformation of uterine spiral arteries during trophoblast invasion is a crucial contributing factor to FGR. The normal transformation of the spiral arteries into low resistance, dilated vessels can be assessed by calculating the RI and PI of the uterine arteries with the help of Ultrasound Doppler. In the future, combining biochemical markers, i.e., H-hCG levels with ultrasound markers such as uterine artery RI and PI, may provide valuable insights into the mechanisms of FGR and serve as a reliable first trimester screening test for FGR. This method of first trimester screening could be viable and simple to implement. H-hCG could replace free β-hCG that is already routinely used in the first trimester screening for Down’s Syndrome, as studies have shown that H-hCG and free β-hCG have similar performance in Down syndrome screening protocols [32,59,60]. To determine H-hCG levels in the blood of pregnant women in the first trimester, an electrochemiluminescence assay, which uses a H-hCG specific antibody, B152, can be used, as this method has already been implemented and is commercially available [61,62]. To our knowledge there are no prospective studies about the correlation between H-hCG and FGR.

Moving further, H-hCG could be administered during implantation and early placental formation to stimulate trophoblast invasion and reduce pregnancy failure and adverse outcomes due to placental dysfunction, including FGR and maybe preeclampsia. The concept of using H-hCG as a therapeutic method is not ground breaking, as “regular” hCG administration is already used in some IVF protocols to increase success rate, but H-hCG may prove to be more effective in promoting successful implantation [63,64,65]. Large-scale production of H-hCG as a commercially available drug is still a matter to be resolved, as there are important steps and procedures to be accomplished. By comparison, one way would be to produce hCG by recombinant DNA techniques. In the case of commercially available recombinant human Chorionic Gonadotropin (Ovidrel), the hCG molecule is obtained by using genetically modified Chinese Hamster Ovary cells that secrete it into the cell culture medium, and afterwards it is purified so that the final product has consistent biological activity and characteristics [66]. Another source of obtaining hCG is from the urine of pregnant females. The commercially chorionic gonadotropin injection, Pregnyl, uses this method and has urine-sourced purified hCG that is standardized by a biological assay procedure [67]. In the case of a commercial drug, the source of H-hCG would most likely be recombinant H-hCG obtained using recombinant DNA techniques and large-scale cell cultures, as this method could use extravillous cytotrophoblast cells that specifically produce the hyperglycosylated variant of hCG. Obtaining H-hCG from placentas or other biological products (e.g., urine) is less feasible, as the concentration is generally small compared to “regular” hCG. Regardless of the source, it is important that at the end of the process of production, a stable, standardized molecule of H-hCG is obtained and that its biological activity is consistent and predictable.

## 7. Conclusions

To summarize, H-hCG appears to have a critical role in facilitating cytotrophoblast invasion, which is essential for successful pregnancy implantation and placental development. If cytotrophoblast invasion is impaired, it can result in inadequate transformation of spiral arteries, which in turn leads to defective placental perfusion. The fetus is then subjected to reduced nutrient and oxygen supply, resulting in growth restriction as an adaptive response to the harsh intrauterine environment. Understanding the function of H-hCG in the evolution of the placenta might unveil new ways to manage and treat fetal growth restriction.

## Data Availability

Not applicable.
